# The Longitudinal Relationship Between Perceived Discrimination and Prosocial Behaviors: The Roles of Self-Esteem and Coping Styles

**DOI:** 10.3390/bs16020172

**Published:** 2026-01-26

**Authors:** Tingyu Gu, Xiaosong Gai, Tianyue Wang

**Affiliations:** 1 School of Psychology, Northeast Normal University, 5268 Renmin Street, Changchun 130024, China; guty577@nenu.edu.cn (T.G.); wangty015@nenu.edu.cn (T.W.); 2 Research Center of Mental Health Education in Northeast Normal University, Key Research Institute of Humanities and Social Science in Universities in Jilin Province, Northeast Normal University, Changchun 130024, China; 3 Changchun City Yangzheng Senior High School, No. 399 Fucheng Road, Changchun 130000, China

**Keywords:** perceived discrimination, self-esteem, prosocial behaviors, positive and negative coping styles, high school students

## Abstract

Although previous studies have established a link between perceived discrimination and negative adolescent outcomes, potential mediating and moderating factors—specifically, the mediating role of self-esteem and the distinct moderating roles of positive and negative coping styles—remain underexplored. This longitudinal study aimed to examine whether adolescents’ perceptions of discrimination directed toward themselves or their classmates predict their prosocial behaviors through the mediating role of self-esteem and whether positive and negative coping styles moderate this pathway. A total of 531 junior high school students (M_age_ = 15.73, SD_age_ = 0.67, 47.83% males) from Changchun, Jilin Province, China, completed measures of perceived discrimination, self-esteem, prosocial behaviors, and coping styles across three time points. Higher levels of perceived discrimination at T1 were associated with fewer prosocial behaviors at T3, and this relationship was mediated by reduced self-esteem at T2. Moreover, both positive and negative coping styles at T1 served as moderators. Positive coping moderated the negative effects of perceived discrimination on both self-esteem and prosocial behaviors, while negative coping moderated the positive association between self-esteem and prosocial behaviors. These findings underscore the distinct role of perceived discrimination, self-esteem, and coping styles in shaping adolescent prosocial development and offer valuable implications for educational interventions aimed at fostering prosociality.

## 1. Introduction

Prosocial behaviors, defined as voluntary actions intended to benefit others (Eisenberg, 1986; Eisenberg & Mussen, 1989; Labroo & Goldsmith, 2021; W. Zhang et al., 2023), encompass a range of activities such as helping, sharing, cooperating, and donating (Liang et al., 2016; Roux et al., 2015). These behaviors represent a crucial component of adolescent development and are fundamental to healthy social functioning. Evidence suggests that prosociality can mitigate the negative psychological effects of stress (Lazar & Eisenberger, 2022), promote health levels (Peacock, 2022), foster a sense of meaning in life (Q. Liu et al., 2020), and facilitate the maintenance of positive interpersonal relationships (H. Zhao et al., 2018). Given these significant benefits, identifying the factors that influence and promote prosocial behaviors in adolescents is an important area of inquiry.

This study focuses on perceived discrimination as a key antecedent, self-esteem as a potential mediator, and coping styles as crucial moderators in shaping adolescent prosocial outcomes. We examine perceived discrimination because it represents a pervasive social stressor that may directly undermine the motivational and emotional foundations of helping others. Self-esteem is investigated as a mediator, given its central role in an adolescent’s self-concept and its established links to both social perception and behavioral engagement. Furthermore, we explore the moderating role of coping styles to understand how different strategies for managing discrimination—whether through positive engagement or negative disengagement—may either buffer or exacerbate its effects on self-esteem and, ultimately, prosocial behavior. This integrated, moderated mediation approach aims to provide a nuanced understanding of the conditions under which discrimination impacts prosocial development, thereby informing more targeted and effective interventions.

### 1.1. Perceived Discrimination and Prosocial Behaviors

Perceived discrimination represents a potentially significant antecedent to prosocial behaviors among adolescents and college students (Aydinli-Karakulak et al., 2021; Davis et al., 2022; H. Li et al., 2011; Partovi et al., 2025; Quan et al., 2024). It is defined as an individual’s belief that they or their group have been subjected to unfair or prejudicial treatment (X. Liu et al., 2013).

According to the Social Support Threat Model, traumatic or stigmatizing experiences—such as perceived discrimination—can undermine interpersonal relationships. When individuals feel discriminated against, they may withdraw from social interactions, thereby impairing the quality of their relationships (Fan et al., 2013; Prelow et al., 2006). This model implies that discrimination may negatively influence behavioral outcomes such as prosociality. In contrast, other theoretical perspectives propose that adverse experiences can sometimes foster greater empathy and concern for others, leading to increased social outreach (Staub & Vollhardt, 2008). From this standpoint, perceived discrimination may, under certain conditions, enhance individuals’ motivation to engage in prosocial acts.

Empirical evidence regarding the association between perceived discrimination and prosocial behaviors remains inconsistent. Several cross-sectional studies have reported a positive relationship. For example, Davis et al. (Davis et al., 2022) surveyed 1527 Latinx college students and found that perceptions of bias toward one’s ethnic group were positively associated with altruistic prosocial behaviors. Similarly, Aydinli-Karakulak et al. (2021), in a study of 195 Turkish-origin adolescents in Bulgaria, observed that perceived cultural discrimination was positively linked to prosocial behaviors. However, these studies focus exclusively on racial or cultural discrimination. It remains unclear whether more directly experienced discrimination—such as that targeting oneself or one’s peers—relates to prosocial outcomes in a similar way.

In contrast, other investigations focusing on perceptions of discrimination directed toward oneself or one’s classmates have demonstrated a significant negative correlation with prosocial behaviors. Cross-sectional research conducted with Chinese adolescents, for instance, has revealed that such direct experiences of discrimination are negatively associated with prosocial behavior (H. Li et al., 2011). Longitudinal evidence further substantiates this pattern. Partovi et al. (2025), in a study tracking 547 U.S. Latino/a adolescents from eighth through ninth grade using cross-lagged panel analyses, found that increased perceptions of school discrimination from peers and teachers predicted subsequent decreases in emotional prosocial behaviors. Similarly, longitudinal research in the Chinese context has confirmed that perceived discrimination at an initial time point negatively predicts later prosocial behavior among adolescents (Quan et al., 2024).

The present study focused specifically on Chinese adolescents’ perceptions of discrimination directed toward themselves or their classmates, rather than racial discrimination specifically. Building on the empirical evidence summarized above, which suggests that such personal experiences of discrimination negatively predict prosocial behaviors, this research aims to identify the longitudinal mediating processes and moderating factors between these two variables. Examination of this issue can provide valuable insights for educators seeking to develop effective interventions aimed at mitigating these adverse effects and fostering prosocial development among adolescents.

### 1.2. Perceived Discrimination and Self-Esteem

Perceived discrimination significantly influences self-esteem among adolescents and college students (Grossman & Liang, 2008; Q. Li et al., 2024; Ruck et al., 2019; Zeiders et al., 2013; J. Zhang et al., 2025). Self-esteem, defined as the affective evaluation of one’s own worth formed through socialization (X. Zhang et al., 2015), may be particularly vulnerable to discriminatory experiences.

According to Symbolic Interactionist Theory, persistent exposure to differential or unfair treatment leads individuals to internalize negative external evaluations, thereby fundamentally threatening their sense of self-worth (Grossman & Liang, 2008). This theoretical framework suggests that perceived discrimination likely undermines self-esteem through repeated negative social mirroring. This process is well-documented in international adolescent development literature, where perceived discrimination is consistently linked to lower global self-esteem across diverse ethnic and national groups (Ruck et al., 2019; Zeiders et al., 2013).

Empirical investigations generally support this proposition, though some inconsistencies exist. Multiple cross-sectional studies with Chinese populations have demonstrated significant negative correlations between various forms of discrimination perception and self-esteem (Bao et al., 2016; X. Liu & Shen, 2010; Sun & Fu, 2024; J. Zhang et al., 2025). Longitudinal evidence further provides support (Q. Li et al., 2024; Q. Xie et al., 2023). For instance, Q. Xie et al. (2023) conducted a four-wave study with Chinese college students and found that prior discrimination consistently negatively predicted subsequent self-esteem. Nevertheless, some studies, particularly those conducted in Western contexts—such as Corning’s (2002) research with European American women—have reported non-significant associations.

### 1.3. Self-Esteem and Prosocial Behaviors

Adolescent prosocial behaviors are closely linked to self-esteem levels. Substantial cross-sectional evidence demonstrates a significant positive correlation between self-esteem and prosocial behaviors across adolescent and university populations (L. Chen & Guan, 2025; Huang et al., 2023; Luo et al., 2025; Yang et al., 2025; X. Zhang et al., 2025; M. Zhao et al., 2025a). Longitudinal studies further establish the predictive relationship between these constructs (Fu et al., 2017; Padilla-Walker et al., 2018; M. Zhao et al., 2025b; Zhu et al., 2023). For example, Fu et al. (2017) conducted a longitudinal panel study with 681 U.S. adolescents, revealing that self-esteem was associated with subsequent prosocial behavior toward strangers, while earlier prosocial behavior also enhanced subsequent self-esteem. Similarly, M. Zhao et al. (2025b) identified self-esteem at age 14 as a significant mediator in the pathway from leisure activities at age 14 to prosocial behavior at age 17.

Building upon the established literature, a conceptual model can be logically derived: adolescents’ perceptions of discrimination directed toward themselves or their classmates negatively predict prosocial behaviors, while discrimination simultaneously undermines self-esteem, which in turn is a known predictor of prosocial development. This pattern suggests that self-esteem may serve as a longitudinal mediating variable through which perceived discrimination ultimately predicts adolescents’ prosocial functioning. Testing this mediated pathway constitutes one of the primary aims of the present study.

### 1.4. Possible Moderating Effects of Coping Styles

Coping encompasses the cognitive and behavioral strategies employed to manage, reduce, or tolerate the demands of stressful situations (Folkman & Moskowitz, 2004). These strategies are frequently categorized into positive (e.g., problem-focused or engagement coping) and negative (e.g., emotion-focused or disengagement coping) styles (Kartalova-O’Doherty & Doherty, 2007). Such coping styles may function as moderators in the mediating pathway from perceived discrimination to prosocial behaviors via self-esteem.

According to the Phenomenological Variant of Ecological Systems Theory (Spencer et al., 2003), supportive resources can buffer the adverse effects of contextual challenges like discrimination. From this perspective, positive coping could mitigate the detrimental impact of perceived discrimination on self-esteem and subsequent prosocial behaviors. Empirical evidence partially supports this view; for instance, F. Chen et al. (2010) found that positive coping moderated the relationship between stress and internalizing problems among Chinese primary school students, suggesting its potential protective role.

Conversely, because discrimination constitutes a systemic and often intractable stressor, active coping efforts may paradoxically exacerbate distress. When individuals invest significant effort into managing discrimination yet see little change, they may experience frustration, helplessness, and psychological depletion (Fischer & Shaw, 1999). This could further erode self-esteem and diminish the emotional resources necessary for prosocial action. Thus, positive coping may in some cases amplify—rather than alleviate—the negative effects of perceived discrimination. Given these opposing theoretical possibilities, the direction of the moderating effect of positive coping remains an open empirical question.

Emerging evidence further suggests a potential moderating role for negative coping styles in the relationship between perceived discrimination and psychological and behavioral outcomes. For instance, Hooberman et al. (2010) demonstrated that emotion-focused coping (i.e., negative coping) significantly moderated the association between cognitive appraisal variables and PTSD symptomatology, often exacerbating symptom severity. Given that self-esteem constitutes a fundamental cognitive appraisal variable, negative coping may similarly moderate its relationship with behavioral outcomes such as prosocial behaviors. Research within organizational contexts found no significant moderating effect of negative coping on the relationship between work stress and mental health among urban management enforcement officials (H. Chen et al., 2010). Considering that perceived discrimination represents a significant stressor and self-esteem constitutes a core component of mental health (H. Li & Mei, 2002; Taylor & Brown, 1988), it remains plausible that negative coping may not significantly moderate the discrimination-self-esteem relationship. Nevertheless, the potential moderating effects of negative coping throughout the pathway from perceived discrimination to prosocial behavior via self-esteem warrant empirical investigation.

### 1.5. The Present Study

This study was designed to longitudinally test an integrated theoretical model examining how perceived discrimination influences adolescent prosocial development. Specifically, we aimed to investigate why and under what conditions this influence occurs by proposing self-esteem as a core mediator and coping styles as key boundary conditions. We hypothesized that discrimination negatively impacts prosocial behaviors not only directly but also indirectly by undermining self-esteem—an internal psychological resource essential for social engagement. Furthermore, we posited that this process is not uniform; the strength of these relationships likely depends on how adolescents cope with stress. Positive coping (e.g., problem-solving) was expected to buffer the harmful effects of discrimination, whereas negative coping (e.g., avoidance) was anticipated to weaken the protective role of self-esteem on prosocial action. Thus, this study had two primary objectives: first, to longitudinally investigate whether adolescents’ perceptions of discrimination directed toward themselves or their classmates predict their prosocial behaviors through the mediating role of self-esteem; and second, to examine whether positive and negative coping styles moderate this mediating pathway.

## 2. Materials and Methods

### 2.1. Participants and Procedure

A cluster sampling method was employed to recruit participants from a high school in Changchun, Jilin Province, China. The study utilized a three-wave longitudinal design with a four-month interval between adjacent assessments. The data collection for the three waves was conducted online in the school computer laboratory under teacher supervision, resulting in minimal participant attrition. To ensure data completeness, the online system prevented submission until all questionnaire items were answered, resulting in no missing data. The study began with an initial sample of 546 participants at Time 1 (T1), when measures of perceived discrimination and coping styles were completed. At Time 2 (T2), 540 participants provided self-esteem data, and at Time 3 (T3), 531 participants completed the prosocial behavior measures. The final longitudinal sample thus consisted of 531 students (254 males, 277 females) with a mean age of 15.73 years (SD = 0.67) at baseline. This study was rigorously reviewed and approved by the Ethics Review and Research Committee of the School of Psychology, Northeast Normal University (2023068). The study was conducted in accordance with the Declaration of Helsinki (as revised in 2013). Informed consent was obtained from all participants and their parents prior to data collection.

### 2.2. Measures

#### 2.2.1. Perceived Discrimination

Perceived discrimination was assessed at T1 using the Perceived Discrimination Scale (PDS) (Shen et al., 2009). This 6-item instrument comprises two subscales: individual discrimination perception (3 items; e.g., “I feel like I’ve been treated unfairly”) and group discrimination perception (3 items; e.g., “Other students from families like mine have been treated unfairly”). Participants responded on a 5-point Likert scale ranging from 1 (completely disagree) to 5 (completely agree). In the present study, Cronbach’s alpha was 0.88 for the total scale, 0.80 for the individual discrimination subscale, and 0.88 for the group discrimination subscale.

#### 2.2.2. Self-Esteem

Self-esteem was measured at T2 using the Chinese version of the Rosenberg Self-Esteem Scale (RSES) (Ji & Yu, 1999; Rosenberg, 1965; Wang et al., 1999). The scale consists of 10 items (e.g., “I feel that I have many good qualities”). Responses were recorded on a 4-point Likert scale. Six positively worded items were scored from 1 (strongly disagree) to 4 (strongly agree), while four negatively worded items were reverse-scored. The total scale demonstrated good internal consistency in this study, with a Cronbach’s alpha of 0.88.

#### 2.2.3. Prosocial Behaviors

Prosocial behaviors were assessed at T3 using the Chinese adaptation (Du et al., 2006) of the Prosocial Behavior Scale from the Strengths and Difficulties Questionnaire (SDQ) (Goodman, 2001). The scale contains 5 items (e.g., “I often volunteer to help others”). Responses were recorded on a 3-point Likert scale ranging from 0 (completely inconsistent) to 2 (completely consistent). Items were averaged to create an average score ranging from 0 to 2, with higher scores indicating greater prosocial tendencies. All items were positively keyed and no reverse scoring was required for this scale. In the current sample, the scale showed excellent internal consistency, with a Cronbach’s alpha of 0.94.

#### 2.2.4. Coping Styles

Coping styles were assessed at T1 using the Simplified Coping Style Scale (SCSS) (Y. Xie, 1998). This 20-item instrument comprises two subscales: positive coping styles (12 items; e.g., “Seeking advice from relatives, friends, or classmates”) and negative coping styles (8 items; e.g., “Relying on others to solve problems”). Participants indicated the frequency with which they adopt each strategy on a 4-point Likert scale ranging from 0 (not adopted) to 3 (frequently adopted). In this study, Cronbach’s alpha was 0.81 for the total scale, 0.84 for the positive coping subscale, and 0.76 for the negative coping subscale.

### 2.3. Analytical Strategy

Analyses were conducted using SPSS 27.0. First, descriptive statistics (means and standard deviations) and correlations among the key study variables were computed. Second, the PROCESS macro v4.2, developed by Hayes (2018), was utilized to evaluate two models: a longitudinal mediation model and a longitudinal moderated mediation model. The longitudinal mediation model (Model 4 of the PROCESS macro) examined whether self-esteem at T2 mediated the relationship between perceived discrimination at T1 and prosocial behaviors at T3. The moderated mediation model (Model 59 of the PROCESS macro) further explored whether this mediating pathway was moderated by positive and negative coping styles at T1.

## 3. Results

### 3.1. Common Method Bias Test

The Harman single-factor test was employed to assess common method deviation. Factor analysis identified 8 factors with eigenvalues greater than 1, with the primary factor explaining 22.86% of the total variance—well below the 40% threshold commonly used to suggest significant common method bias. Thus, this research did not appear to be affected by substantial common method bias (Zhou & Long, 2004).

### 3.2. Descriptive Statistics and Correlation Analyses

Preliminary analyses examined the descriptive statistics and correlations among all study variables, as presented in Table 1.

Perceived discrimination at T1 was negatively correlated with positive coping styles at T1, self-esteem at T2, and prosocial behaviors at T3, and positively correlated with negative coping styles at T1. Self-esteem at T2 was positively correlated with positive coping styles at T1 and prosocial behaviors at T3, and negatively correlated with negative coping styles at T1. Prosocial behaviors at T3 were positively correlated with positive coping styles at T1. A significant positive correlation was also observed between positive and negative coping styles. These initial findings supported the need for further analysis of the complex relationships proposed in our theoretical model.

### 3.3. Mediation Model Test

Prior to conducting the primary regression-based analyses, we examined the key statistical assumptions. First, multicollinearity was assessed using variance inflation factors (VIF) for all predictor variables in the regression models. All VIF values were below 2 (range: 1.12–1.38), well under the conventional threshold of 5, indicating no substantial multicollinearity concerns. Second, linearity and homoscedasticity assumptions were evaluated by examining scatterplots of standardized residuals against standardized predicted values. Visual inspection revealed no systematic patterns, supporting the assumptions of linear relationships and constant variance. Third, normality of residuals was assessed using P-P plots and histograms of standardized residuals. The residual distributions showed acceptable approximation to normality, with skewness and kurtosis values within recommended bounds (all < |2|). Fourth, we conducted influence diagnostics using Cook’s distance and leverage values. No influential outliers were identified (all Cook’s D values < 0.07, all leverage values < 0.07). Finally, given that moderated mediation analyses can be sensitive to distributional assumptions, we employed bias-corrected bootstrapping with 5000 resamples for all indirect effect tests. This non-parametric approach provides robust confidence interval estimation without relying on strict normality assumptions.

The data structure involved students nested within classrooms. To account for the potential non-independence of observations arising from this clustering, we incorporated classroom membership as a statistical control. Specifically, dummy-coded variables representing each classroom (with one classroom as the reference group) were included as covariates in all primary analyses using the PROCESS macro. This fixed-effects approach controls for all time-invariant differences between classrooms.

**Figure 1:**
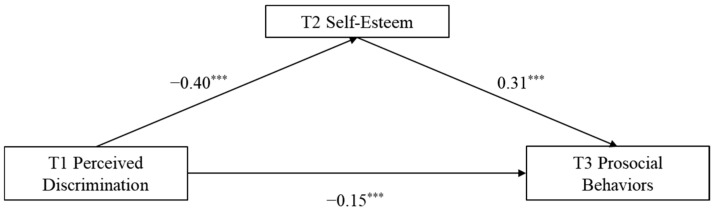
The mediating role of self-esteem. Note: Class, gender, and age as control variables; * *p* < 0.05, ** *p* < 0.01, *** *p* < 0.001 (two-tailed).

A longitudinal mediation model was tested using the PROCESS macro to examine the relationships among perceived discrimination at T1, self-esteem at T2, and prosocial behaviors at T3. The results are shown in Figure 1.

The results indicated that self-esteem at T2 partially mediated the relationship between perceived discrimination at T1 and prosocial behaviors at T3. Specifically, higher levels of perceived discrimination at T1 predicted lower self-esteem at T2, which in turn predicted fewer prosocial behaviors at T3. Furthermore, perceived discrimination at T1 also exerted a significant direct effect on prosocial behaviors at T3. The direct effect was −0.15, 95% CI [−0.24, −0.07]. The indirect effect through self-esteem was −0.12, 95% CI [−0.17, −0.08]. The mediating effect of self-esteem accounted for 44.76% of the total effect.

### 3.4. Moderated Mediation Model Test

A longitudinal moderated mediation model was tested to examine whether coping styles at T1 moderate the mediating pathway from perceived discrimination at T1 to prosocial behaviors at T3 via self-esteem at T2. The results indicated significant moderating effects for both positive and negative coping styles.

As shown in Table 2, positive coping styles significantly moderated two paths in the mediation model. The interaction of perceived discrimination and positive coping styles negatively predicted both self-esteem at T2 (β = −0.09, *p* < 0.05, 95% CI [−0.18, −0.01]) and prosocial behaviors at T3 (β = −0.10, *p* < 0.05, 95% CI [−0.20, −0.003]). The conditional indirect effects of perceived discrimination on prosocial behaviors via self-esteem were negative and significant at low (b = −0.06, 95% CI [−0.11, −0.03]), mean (b = −0.08, 95% CI [−0.12, −0.04]), and high (b = −0.08, 95% CI [−0.16, −0.02]) levels of positive coping. Johnson-Neyman analyses were conducted to delineate the regions of significance for the moderated paths. Two regions are reported, corresponding to the two paths moderated by positive coping. For the first stage of the mediation (the effect of T1 perceived discrimination on T2 self-esteem), the effect was statistically significant for the vast majority of the sample—specifically, when T1 positive coping scores were greater than −1.85 standard deviations below the mean. This condition was met for 97.93% of participants, indicating that the initial link in the indirect pathway is robust across nearly all observed levels of positive coping. For the direct effect (the effect of T1 perceived discrimination on T3 prosocial behavior, controlling for T2 self-esteem), this direct effect was significant when T1 positive coping scores were greater than −0.59 standard deviations below the mean, a condition met for 73.63% of participants. This suggests that the direct pathway from discrimination to prosocial behavior is active primarily at moderate-to-high levels of positive coping.

Table 3 demonstrates that negative coping styles moderated the relationship between self-esteem and prosocial behaviors. The interaction of self-esteem and negative coping negatively predicted prosocial behaviors (β = −0.10, *p* < 0.01, 95% CI [−0.18, −0.03]), indicating that the protective effect of self-esteem on prosocial behaviors was attenuated at higher levels of negative coping. The conditional indirect effects of perceived discrimination on prosocial behaviors via self-esteem were negative and significant at low (b = −0.18, 95% CI [−0.25, −0.11]), mean (b = −0.12, 95% CI [−0.17, −0.08]), and high (b = −0.08, 95% CI [−0.14, −0.03]) levels of negative coping. To precisely identify the boundary condition of the moderated mediation, we conducted a Johnson-Neyman analysis. The results specified that the indirect effect of T1 perceived discrimination on T3 prosocial behavior via T2 self-esteem was statistically significant when T1 negative coping scores were below 1.63. This critical value demarcates a clear region of significance: the indirect effect was sustained for the vast majority of participants (92.47%) who reported low to moderate levels of negative coping, but it became non-significant for those with very high levels of negative coping (scores above 1.63, representing 7.53% of the sample).

**Figure 2:**
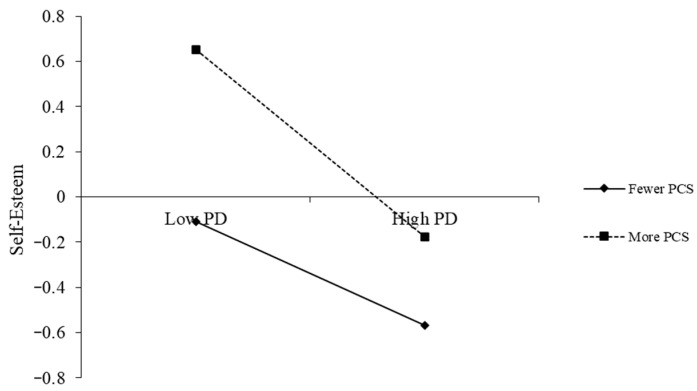
The moderating effect of positive coping styles on the relationship between perceived discrimination and self-esteem. Note: Class, gender, and age as control variables; PD = Perceived Discrimination, PCS = Positive Coping Styles.

**Figure 3:**
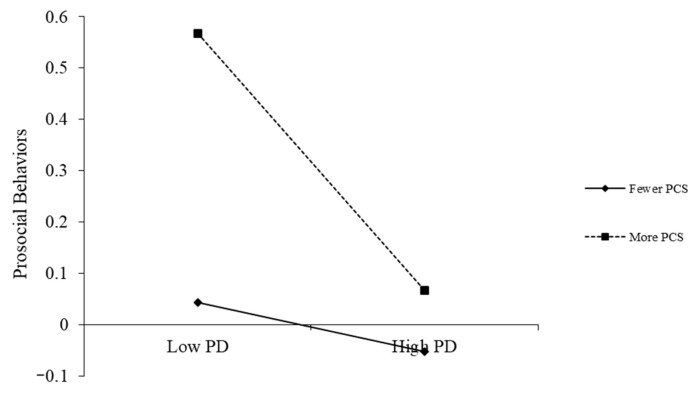
The moderating effect of positive coping styles on the relationship between perceived discrimination and prosocial behaviors. Note: Class, gender, and age as control variables; PD = Perceived Discrimination, PCS = Positive Coping Styles.

**Figure 4:**
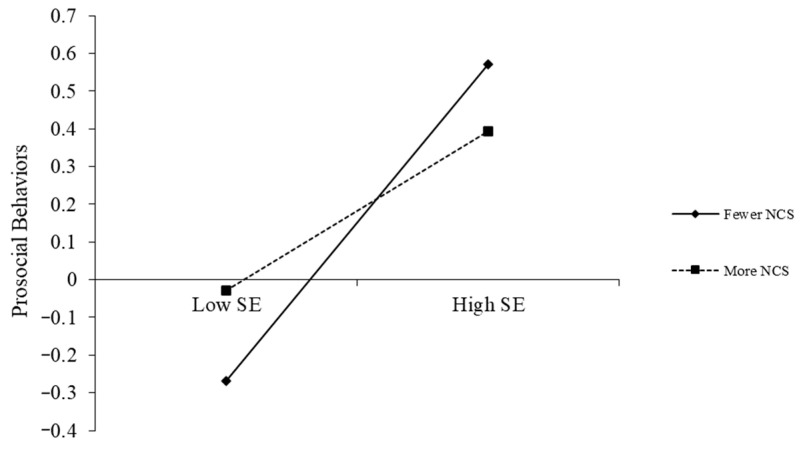
The moderating effect of negative coping styles on the relationship between self-esteem and prosocial behaviors. Note: Class, gender, and age as control variables; SE = Self-Esteem, NCS = Negative Coping Styles.

To clarify the nature of the significant interaction effects, simple slope analyses were conducted. The findings are presented in Figure 2, Figure 3 and Figure 4.

As illustrated in Figure 2, students who employed more positive coping styles generally reported higher self-esteem across all levels of perceived discrimination. However, the negative association between perceived discrimination and self-esteem was stronger among students with higher levels of positive coping (simple slope = −0.42, *t* = −5.55, *p* < 0.001) compared to those with lower levels (simple slope = −0.23, *t* = −4.66, *p* < 0.001).

Figure 3 demonstrates that the relationship between perceived discrimination and prosocial behaviors was moderated by positive coping styles. Students who employed more positive coping styles generally reported more prosocial behaviors across all levels of perceived discrimination. For students reporting lower positive coping, perceived discrimination did not significantly predict prosocial behaviors (simple slope = −0.05, *t* = −0.91, *p* = 0.36 > 0.05). In contrast, among students with higher positive coping, greater perceived discrimination predicted significantly fewer prosocial behaviors (simple slope = −0.25, *t* = −3.03, *p* = 0.003 < 0.01).

As shown in Figure 4, negative coping styles moderated the association between self-esteem and prosocial behaviors. While self-esteem positively predicted prosocial behaviors at both levels of negative coping, this relationship was stronger for students reporting lower negative coping (simple slope = 0.42, *t* = 6.99, *p* < 0.001) than for those reporting higher negative coping (simple slope = 0.21, *t* = 3.73, *p* < 0.001).

## 4. Discussion

### 4.1. The Mediating Role of Self-Esteem

Our findings demonstrate that adolescents’ perceptions of discrimination directed toward themselves or their classmates at T1 significantly predicted decreased prosocial behaviors at T3. This result aligns with previous research examining discrimination directed toward oneself or one’s social group (H. Li et al., 2011; Partovi et al., 2025; Quan et al., 2024), reinforcing the conclusion that exposure to discriminatory treatment undermines adolescents’ propensity for prosocial action. From an applied perspective, this finding underscores the importance of cultivating inclusive educational environments to foster prosocial development. However, merely reducing discrimination, while crucial, may be insufficient. It is also necessary to examine the mediating variables in the relationship between perceived discrimination and prosocial behaviors in order to take measures from multiple perspectives to improve students’ prosocial behaviors.

Our findings regarding the relationship between perceived discrimination and self-esteem revealed that T1 discrimination significantly predicted lower self-esteem at T2. This result aligns with numerous previous studies (Bao et al., 2016; Q. Li et al., 2024; X. Liu & Shen, 2010; Sun & Fu, 2024; Q. Xie et al., 2023; J. Zhang et al., 2025), though inconsistencies exist in the literature (Corning, 2002; Tom, 2006). These discrepant findings may be attributed to methodological factors, including specific sample characteristics and cross-sectional designs. For instance, Corning’s (2002) cross-sectional study with primarily European American women found no significant association. The longitudinal design of the present study provides stronger evidence for the temporal precedence of discrimination in predicting self-esteem deterioration. Furthermore, our results confirmed that self-esteem at T2 significantly positively predicted prosocial behaviors at T3, consistent with established literature (L. Chen & Guan, 2025; Fu et al., 2017; Huang et al., 2023; Luo et al., 2025; Padilla-Walker et al., 2018; Yang et al., 2025; X. Zhang et al., 2025; M. Zhao et al., 2025a; M. Zhao et al., 2025b; Zhu et al., 2023).

The longitudinal mediation analysis demonstrated that self-esteem partially mediated the relationship between perceived discrimination and prosocial behaviors. Specifically, adolescents who experienced greater discrimination developed lower self-esteem, which in turn led to reduced prosocial behaviors. This can be understood through theoretical frameworks that posit that individuals who experience discrimination may internalize negative external evaluations (Crocker & Major, 1989). These internalized evaluations can form negative self-concepts, depleting the psychological resources that would otherwise be available for helping others (Baumeister et al., 2003). These findings extend previous research by elucidating the longitudinal pathways through which discrimination affects prosocial development. From an educational perspective, these results highlight the importance of implementing programs designed to enhance self-esteem. Such programs could serve as a potential buffer against the negative effects of discrimination on prosocial behavior. Interventions targeting self-concept improvement may help mitigate the adverse consequences of discriminatory experiences on adolescents’ social functioning.

### 4.2. The Moderating Effects of Positive and Negative Coping Styles

The moderated mediation analyses revealed complex patterns concerning the role of coping styles. Positive coping styles at T1 moderated the relationships between perceived discrimination at T1 and both self-esteem at T2 and prosocial behaviors at T3. Simple slope analyses indicated that students employing more positive coping strategies generally maintained higher levels of both self-esteem and prosocial behavior across all levels of discrimination. This finding aligns with the Stress Coping Theory (Lazarus & Folkman, 1984), suggesting that positive coping serves as a valuable personal resource, potentially through enhancing perceived control, strengthening social support networks, and improving emotional regulation capabilities (Taylor & Stanton, 2007).

However, contrary to conventional expectations, the simple effects analyses also found that the negative association between perceived discrimination and both outcomes was stronger among students who frequently used positive coping strategies. This pattern suggests that while positive coping is generally beneficial, it may paradoxically exacerbate the detrimental effects of discrimination. From a theoretical standpoint, this finding may reflect the potential costs of “engagement coping” under uncontrollable stressors (Compas et al., 2014). While approach-oriented strategies like problem-solving are typically adaptive, they may become maladaptive when applied to chronic, systemic stressors like discrimination that are not easily resolved through individual effort. Persistent engagement with an uncontrollable stressor can lead to repeated failure experiences, heightened frustration, and emotional exhaustion (Miller & Kaiser, 2001), which could explain the steeper decline in self-esteem and prosociality among high copers. This aligns with the responsiveness hypothesis, where the same coping strategy that benefits general well-being may increase sensitivity to specific, uncontrollable adversities (Forsythe & Compas, 1987). In addition, another alternative explanation is that coping efficacy is context-dependent. The benefits of a coping strategy cannot be judged in isolation from the characteristics of the stressor. Discrimination, as a pervasive and identity-relevant threat, may undermine the very mechanisms—such as perceived control or trust in social systems—upon which positive coping strategies often rely. Therefore, what is generally a resource may, in this specific context, become a locus of vulnerability.

These findings carry significant implications for educational practice. Promoting generic positive coping (e.g., problem-solving, direct action) without considering stressor controllability could inadvertently increase vulnerability to systemic, identity-based threats like discrimination. Therefore, psychoeducation should help students develop coping flexibility (Bonanno & Burton, 2013). For controllable challenges, “engagement” strategies (problem-solving, seeking instrumental support) remain paramount. For uncontrollable stressors like discrimination, the educational focus should shift toward “accommodation” or impact management strategies (Compas et al., 2014), such as emotional acceptance, cognitive reappraisal of the self (not the event), selective disengagement, and resource preservation. Furthermore, concurrent and paramount efforts must include environmental interventions—such as implementing robust anti-discrimination policies, establishing effective reporting mechanisms (Gregory et al., 2016), and fostering inclusive school climates (Hatzenbuehler, 2016)—to reduce the exposure to the uncontrollable stressor itself. This systemic work ensures that students’ coping efforts, particularly engagement-oriented ones, can eventually operate within a context where they have the potential to yield meaningful change.

Regarding negative coping styles, our results demonstrated their moderating effect on the relationship between self-esteem and prosocial behaviors, thereby extending previous research (Hooberman et al., 2010). The protective effect of self-esteem on prosocial behavior was substantially weakened among students who relied heavily on negative coping strategies. This may be because such strategies continuously consume emotional regulation resources (Joormann & Stanton, 2016), leaving insufficient psychological capacity to recognize others’ needs or engage in prosocial actions (Penner et al., 2005).

This suggests that intervention efforts should extend beyond self-esteem enhancement to include coping style optimization. For students who frequently use negative coping strategies—even those with high self-esteem—participation in structured, low-social-risk activities may be beneficial. These activities can allow them to experience the positive impact of their behaviors without inducing anxiety, thereby helping them gradually build self-efficacy. This process may, in turn, help modify maladaptive coping patterns and improve prosocial behaviors.

### 4.3. Limitations and Future Directions

Although this study elucidates the mediating and moderating variables of perceived discrimination affecting prosocial behavior, there are several limitations worth considering.

First, the longitudinal design employed in this study, while allowing for tests of temporal precedence and mediation, measured each core variable at only selected time points. This approach does not enable an analysis of intra-individual developmental trajectories or changes in these constructs over the study period. Future research incorporating repeated measures of all variables at each wave would allow for growth curve modeling and a more dynamic understanding of how these processes unfold over time. Furthermore, we did not include measures of self-efficacy, which could provide additional insight into the psychological mechanisms at play. Future research would benefit from measuring coping styles at all waves and incorporating self-efficacy assessments to examine their dynamic roles across the developmental period.

Second, although we identified self-esteem as a mediator and coping styles as moderators, other important psychological and contextual factors likely shape these relationships. In particular, parenting styles represent a potentially influential variable that could affect both the experience of discrimination and the development of coping resources. Future studies should consider incorporating additional variables such as parenting styles, social identity, empathy, and perceived social support to build more comprehensive models of how discrimination affects adolescent development.

Third, the data for this study were all collected from a single school. While this approach helped control for school-level confounding variables, it limits the generalizability of the sample to the broader student population. Future research should include participants from diverse regions and different types of schools to examine the generalizability of the findings.

Fourth, our reliance on self-report measures may introduce common method bias. Utilizing multi-method assessments, including peer nominations or behavioral observations in standardized contexts (Podsakoff et al., 2003), would enhance the validity and robustness of future findings.

Finally, the cultural context of our Chinese sample may limit generalizability. Cultural variations in coping strategies and prosocial norms between collectivist and individualistic societies suggest the value of cross-cultural comparisons to determine the universality or cultural specificity of the observed relationships.

## 5. Conclusions

This longitudinal study yields two primary conclusions regarding the relationship between perceived discrimination and prosocial behaviors in Chinese adolescents. First, perceived discrimination exerts a significant negative influence on subsequent prosocial behaviors, and this relationship is partially mediated by diminished self-esteem. Second, this mediating pathway is moderated by both positive and negative coping styles in distinct ways: positive coping styles moderate the initial stages of the pathway (between discrimination and both self-esteem and prosocial behaviors), while negative coping styles moderate the latter stage (between self-esteem and prosocial behaviors). These findings underscore the complex interplay among environmental stressors, internal psychological resources, and behavioral outcomes. They highlight the need for multifaceted intervention approaches. Such approaches should aim not only to reduce discrimination but also to enhance self-esteem and cultivate adaptive coping strategies.

## Figures and Tables

**Table 1:** Descriptive statistics and correlations for the variables.

Variables	M	SD	1	2	3	4	5
1. T1 Perceived discrimination	1.43	0.56	1				
2. T2 Self-esteem	3.14	0.51	−0.40 ***	1			
3. T3 Prosocial behaviors	1.50	0.52	−0.27 ***	0.36 ***	1		
4. T1 Positive coping styles	1.74	0.56	−0.38 ***	0.42 ***	0.32 ***	1	
5. T1 Negative coping styles	1.01	0.57	0.19 ***	−0.15 ***	−0.08	0.12 **	1

Note: * *p* < 0.05, ** *p* < 0.01, *** *p* < 0.001 (two-tailed).

**Table 2:** The moderating effects of positive coping styles on mediation model.

	Dependent Variable: T2 SE			Dependent Variable: T3 PB		
	β	*SE*	95% CI	β	*SE*	95% CI
Class	0.01	0.01	[−0.01, 0.03]	−0.01	0.01	[−0.03, 0.01]
Gender	−0.09	0.08	[−0.24, 0.06]	−0.21 **	0.08	[−0.36, −0.05]
Age	0.05	0.04	[−0.02, 0.13]	−0.04	0.04	[−0.11, 0.04]
T1 PD	−0.32 ***	0.04	[−0.41, −0.23]	−0.15 **	0.05	[−0.24, −0.05]
T1 PCS	0.29 ***	0.04	[0.21, 0.37]	0.16 ***	0.05	[0.07, 0.25]
T1 PD × T1 PCS	−0.09 *	0.04	[−0.18, −0.01]	−0.10 *	0.05	[−0.20, −0.003]
T2 SE				0.24 ***	0.05	[0.15, 0.33]
T2 SE × T1 PCS				−0.04	0.04	[−0.12, 0.04]
*R* ^2^	0.25			0.20		
*F*	29.77 ***			15.86 ***		

Note: PD = Perceived Discrimination, SE = Self-Esteem, PB = Prosocial Behaviors, PCS = Positive Coping Styles; * *p* < 0.05, ** *p* < 0.01, *** *p* < 0.001 (two-tailed).

**Table 3:** The moderating effects of negative coping styles on mediation model.

	Dependent Variable: T2 SE			Dependent Variable: T3 PB		
	β	*SE*	95% CI	β	*SE*	95% CI
Class	−0.01	0.01	[−0.02, 0.03]	−0.01	0.01	[−0.03, 0.01]
Gender	−0.06	0.08	[−0.22, 0.09]	−0.19 *	0.08	[−0.35, −0.03]
Age	0.06	0.04	[−0.02, 0.13]	−0.05	0.04	[−0.12, 0.03]
T1 PD	−0.39 ***	0.04	[−0.48, −0.31]	−0.15 **	0.05	[−0.24, −0.06]
T1 NCS	−0.08	0.04	[−0.16, 0.004]	0.01	0.04	[−0.07, 0.10]
T1 PD × T1 NCS	0.02	0.04	[−0.05, 0.10]	−0.02	0.04	[−0.11, 0.06]
T2 SE				0.32 ***	0.04	[0.23, 0.40]
T2 SE × T1 NCS				−0.10 **	0.04	[−0.18, −0.03]
*R* ^2^	0.17			0.18		
*F*	17.91 ***			13.96 ***		

Note: PD = Perceived Discrimination, SE = Self-Esteem, PB = Prosocial Behaviors, NCS = Negative Coping Styles; * *p* < 0.05, ** *p* < 0.01, *** *p* < 0.001 (two-tailed).

## Data Availability

The data that support the findings of this study are available from the corresponding author upon reasonable request.
